# Association of Platelets and White Blood Cells Subtypes with Trauma Patients’ Mortality Outcome in the Intensive Care Unit

**DOI:** 10.3390/healthcare9080942

**Published:** 2021-07-26

**Authors:** Ruei-Ti Ke, Cheng-Shyuan Rau, Ting-Min Hsieh, Sheng-En Chou, Wei-Ti Su, Shiun-Yuan Hsu, Ching-Hua Hsieh, Hang-Tsung Liu

**Affiliations:** 1 Department of Surgery, Kaohsiung Chang Gung Memorial Hospital and Chang Gung University College of Medicine, Kaohsiung 833, Taiwan; yaself71@gmail.com; 2 Department of Neurosurgery, Kaohsiung Chang Gung Memorial Hospital and Chang Gung University College of Medicine, Kaohsiung 833, Taiwan; ersh2127@cloud.cgmh.org.tw; 3 Department of Trauma Surgery, Kaohsiung Chang Gung Memorial Hospital and Chang Gung University College of Medicine, Kaohsiung 833, Taiwan; hs168hs168@gmail.com (T.-M.H.); athenechou@gmail.com (S.-E.C.); s101132@adm.cgmh.org.tw (W.-T.S.); ah.lucy@hotmail.com (S.-Y.H.)

**Keywords:** white blood cell, monocyte-to-lymphocyte ratio, neutrophil-to-lymphocyte ratio, platelet-to-lymphocyte ratio, intensive care unit

## Abstract

**Background:** White blood cell (WBC) subtypes have been suggested to reflect patients’ immune-inflammatory status. Furthermore, the derived ratio of platelets and WBC subtypes, including monocyte-to-lymphocyte ratio (MLR), neutrophil-to-lymphocyte ratio (NLR), and platelet-to-lymphocyte ratio (PLR), is proposed to be associated with patient outcome. Therefore, this study aimed to identify the association of platelets and white blood cells subtypes with the mortality outcome of trauma patients in the intensive care unit (ICU). **Method:** The medical information from 2854 adult trauma patients admitted to the ICU between 1 January 2009 and 31 December 2019 were retrospectively retrieved from the Trauma Registry System and classified into two groups: the survivors group (*n* = 2524) and the death group (*n* = 330). The levels of monocytes, neutrophils, lymphocytes, platelets, and blood-drawn laboratory data detected upon patient arrival to the emergency room and the derived MLR, NLR, and PLR were calculated. Multivariate logistic regression analysis was used to determine the independent effects of univariate predictive variables on mortality occurrence. **Result:** The results revealed the patients who died had significantly lower platelet counts (175,842 ± 61,713 vs. 206,890 ± 69,006/μL, *p* < 0.001) but higher levels of lymphocytes (2458 ± 1940 vs. 1971 ± 1453/μL, *p* < 0.001) than the surviving patients. However, monocyte and neutrophil levels were not significantly different between the death and survivor groups. Moreover, dead patients had a significantly lower PLR than survivors (124.3 ± 110.3 vs. 150.6 ± 106.5, *p* < 0.001). However, there was no significant difference in MLR or NLR between the dead patients and the survivors. Multivariate logistic regression revealed that male gender, old age, pre-existing hypertension, coronary artery disease and end-stage renal disease, lower Glasgow Coma Scale (GCS), higher Injury Severity Score (ISS), higher level of lymphocytes and lower level of red blood cells and platelets, longer activated partial thromboplastin time (aPTT), and lower level of PLR were independent risk factors associated with higher odds of trauma patient mortality outcome in the ICU. **Conclusion:** This study revealed that a higher lymphocyte count, lower platelet count, and a lower PLR were associated with higher risk of death in ICU trauma patients.

## 1. Introduction

Complete blood count (CBC) is one of the most frequent examinations to provide information on hemogram distribution in trauma patients. In addition to red blood cells (RBCs), white blood cells (WBCs), platelets, and information on WBC subtypes can be obtained. Several articles have reviewed WBC subtypes and distribution that could reflect patient immune-inflammatory status. The initial WBC count could be a predictor of subsequent bloodstream infection in burn patients [1]. Furthermore, the derived ratio of platelets and WBC subtypes are readily available parameters that might predict outcome [2,3,4,5,6]. For example, the monocyte-to-lymphocyte ratio (MLR) in osteoporosis patients [7], the neutrophil-to-lymphocyte ratio (NLR) and the platelet-to-lymphocyte ratio (PLR) in oncology patients [8,9,10], and the NLR in fracture patients [11] were also reported to be novel predictors of mortality in these specific patient populations. 

The immune-inflammatory response has contributed to outcome determination in critically ill patients [12,13]. Coagulopathy induced by major trauma may affect around one-third of trauma patients [14]. Of trauma patients with critical injury, 45.5% of patients had below-normal platelet response to at least one agonist (“platelet hypofunction”) at admission, and 91.1% had platelet hypofunction during their stay in the intensive care unit (ICU) [15]. The development of trauma-induced coagulopathy would lead to secondary bleeding from a widespread microvascular hemorrhage that was not localized to the site of injury [14]. Many studies have demonstrated that severe trauma may also result in remarkable blood loss and lead to platelet consumption [16,17,18]. Further, lymphocyte levels are related to multiple organ dysfunction syndromes in patients with traumatic injuries [19]. In addition to the number of lymphocytes, the number of the other two types of immune cells (neutrophils and monocytes) can also reflect not only the immune status of patients but also the hematopoietic function of the bone marrow [20]. Both of conditions may impact patient outcome following major trauma. The change of platelet levels and those of the above WBC subtypes would lead to a change of the MLR, NLR, and PLR in trauma patients. Since injuries generally occur accidentally and acutely and since trauma patients are a specific population, it is interesting to determine whether the platelets and the subtypes of the WBC or MLR, NLR, and PLR have been associated with the prognosis of critically injured trauma patients. Therefore, this study aimed to identify the association of platelets and white blood cell subtypes with trauma patient mortality outcome in the intensive care unit.

## 2. Materials and Methods

### 2.1. Study Population 

**Figure 1:**
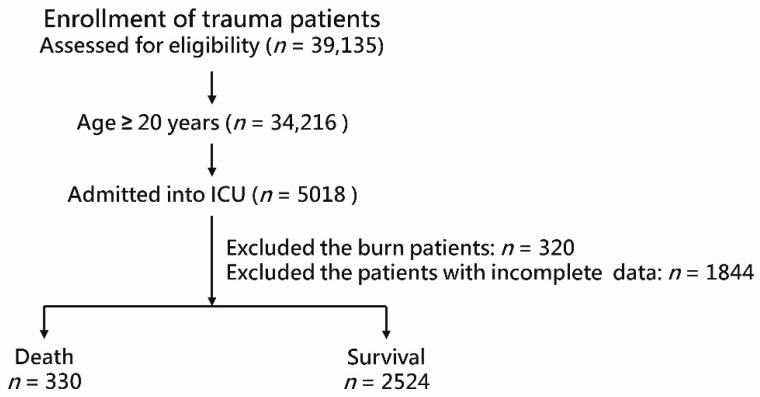
The enrollment of the study population.

This study was approved (approval numbers: 201901582B0 and 202000689B0) by the Institutional Review Board (IRB) of Chang Gung Memorial Hospital, a level I trauma center in southern Taiwan. The requirement for informed consent was waived by the IRB because of the retrospective design of the study. Detailed information on the trauma patients admitted to the ICU, prospectively registered in the Trauma Registry System of the hospital from 1 January 2009 to 31 December 2019, was retrospectively retrieved for analysis. Among the 39,135 enrolled trauma patients during this time period, 34,216 of them were adults, and 5018 adult patients were admitted to the ICU. After excluding patients with burn injuries (*n* = 320) and those with incomplete data (*n* = 1844), 2854 patients were enrolled in the study (Figure 1). Data, including sex, age, comorbidities (cerebrovascular accident (CVA), hypertension (HTN), coronary artery disease (CAD]), congestive heart failure (CHF), diabetes mellitus (DM), and end-stage renal disease (ESRD)), Glasgow Coma Scale (GCS) score, Injury Severity Score (ISS), and in-hospital mortality were retrieved from the Trauma Registry System of the hospital [21,22,23]. The levels of monocytes, neutrophils, lymphocytes, platelets, prothrombin time (PT), activated partial thromboplastin time (aPTT), international normalized ratio (INR), aspartate aminotransferase (AST), alanine aminotransferase (ALT), blood urea nitrogen (BUN), and creatinine (Cr) detected and recorded upon patient arrival to the emergency room were recorded, and the MLR, NLR, and PLR were calculated accordingly. 

### 2.2. Statistical Analysis

Statistical analysis was performed using the IBM SPSS Statistics 23 software (IBM, corporation, Armonk, NY, USA). The patients were classified into two groups: the survivors group (*n* = 2524) and the death group (*n* = 330). Continuous data are expressed as mean ± standard deviation while presenting the GCS and ISS as the median and the interquartile range (IQR, Q1–Q3), respectively. Continuous variables were estimated using Levene’s test for homogeneity of variance and analyzed using one-way analysis of variance with a Games–Howell post hoc test. Categorical variables were analyzed using the chi-square test for the odds ratio and a 95% confidence interval (CI). Multivariate logistic regression analysis was applied to identify the independent effects of univariate predictive variables on mortality in adult trauma patients in the ICU. Based on the identified mortality independent risk factors, a receiver operating characteristic (ROC) curve was applied to plot the relationship between the sensitivity and the 1-specificity. Addionally, the area under the curve (AUC) was calculated to judge the discrimination of the ROC curve. Statistical significance was set at *p* < 0.05.

## 3. Results

### 3.1. Patient Demographics

As shown in Table 1, among the 2854 adult trauma patients in the ICU, males had significantly higher mortality rates than females (69.4% vs 30.6%, *p* = 0.031). The elderly and those with the comorbidities of HTN (44.5% vs. 34.6%, *p* < 0.001), CAD (13.3% vs. 6.6%, *p* < 0.001), and ESRD (9.7% vs. 2.2%, *p* < 0.001) had a higher risk of mortality outcome. A significantly lower GCS score (median (IQR): 5 [3–12] vs. 15 [11–15], *p* < 0.001) and higher ISS (median (IQR): 25 [25–29] vs. 17 [16–24], *p* < 0.001) were observed in the death group than in the survivors group. Under stratification by ISS, significantly fewer dead patients had an ISS of 1–15 and 16–24, and more dead patients had an ISS ≥ 25 than the survivors. Dead patients had a significantly lower level of RBCs (3.9 ± 0.9 vs. 4.3 ± 0.8 millions/μL, *p* < 0.001) and lower platelet counts (175,842 ± 61,713 vs. 206,890 ± 69,006 /μL, *p* < 0.001) but higher lymphocytes levels (2458 ± 1940 vs. 1971 ± 1453 /μL, *p* < 0.001) than the survivors. However, monocyte and neutrophil levels were not significantly different between the death and survivor groups. Furthermore, PLR, but not MLR and NLR, was significantly different between the dead patients and the survivors. Dead patients had a significantly lower PLR than the survivors (124.3 ± 110.3 vs. 150.6 ± 106.5, *p* < 0.001). In addition, the dead patients had significantly higher PT, aPTT, INR, BUN, and Cr than the survivors. However, there was no difference in liver function levels presenting with AST and ALT between the dead and surviving patients. Dead patients had a significantly longer length of hospital stay than the survivors (11.1 days vs. 7.7 days, *p* < 0.001).

### 3.2. Risk Factors for Mortality

Univariate logistic regression analysis (Table 2) showed that the significant risk factors for mortality in adult trauma patients in the ICU were male gender, old age, pre-existing HTN, CAD, ESRD, lower GCS, higher ISS, lower levels of RBCs and platelets, a higher level of lymphocytes, longer bleeing time (PT, aPTT, and INR), and a higher level of BUN, and Cr. Multivariate logistic regression analysis revealed that old age, pre-existing CAD, ESRD, lower GCS, higher ISS, a higher level of lymphocytes, a lower level of RBCs and platelet levels, longer aPTT, and a lower level PLR remained significant independent risk factors for mortality. Among these variables, ESRD was associated with the highest odds (OR, 3.12; 95% CI, 1.32–7.37; *p* = 0.010) of mortality risk, followed by CAD (OR, 1.73; 95% CI, 1.09–2.75; *p* = 0.020). In addition, there were significantly higher odds of mortality risk for lymphocyte and platelet levels (lymphocytes: OR, 1.31; 95% CI, 1.16–1.48; *p* < 0.001; platelets: OR, 0.54; 95% CI, 0.41–0.67; *p* < 0.001), RBC (OR, 0.80; 95% CI, 0.66–0.98; *p* = 0.032), PLR (OR, 0.98; 95% CI, 0.98–0.99; *p* = 0.024), and aPTT (OR, 1.06; 95% CI, 1.03–1.09; *p* < 0.001).

### 3.3. Prediction for Mortality

**Figure 2:**
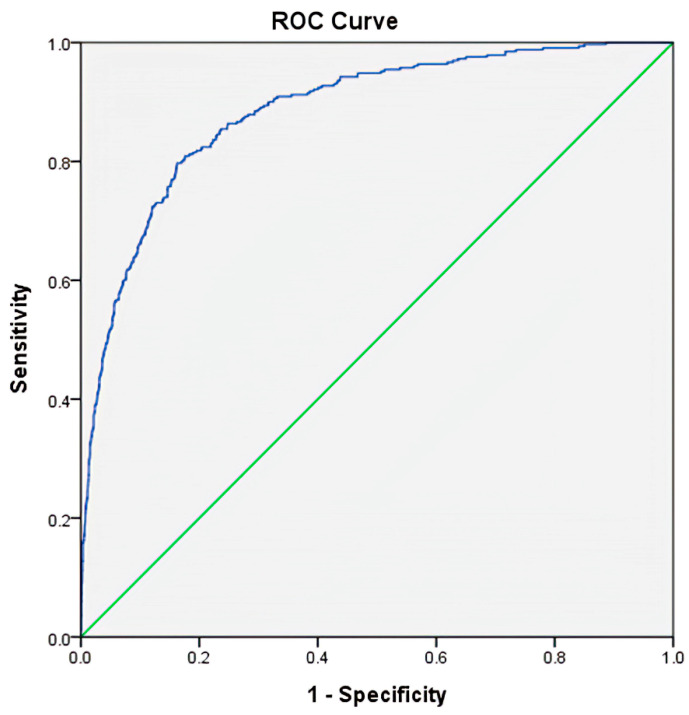
The receiver operating characteristic curves and the area under the curve (AUC) of the selected variables, including age, coronary artery disease (CAD), end-stage renal disease (ESRD), Glasgow Coma Scale (GCS), Injury Severity Score (ISS), activated partial thromboplastin time (aPTT), and platelet-to-lymphocyte ratio (PLR) to predict mortality for adult trauma patients with critical illness.

Furthermore, an ROC was applied to predict mortality outcomes (Figure 2). The values applied for mortality prediction were determined by a regression calculation using the value of −5.680 + 0.424 × Male (yes or no) + 0.039 × Age + 0.597 × CAD (yes or no) + 1.924 × ESRD (yes or no) + −0.243 × GCS + 0.065 × ISS + 0.066 × aPTT + 0.880 × PLR. With the best cutoff point set at 0.130, the AUC was 0.89, and the accuracy, sensitivity, and specificity were 83.3%, 79.7%, and 83.8%, respectively.

## 4. Discussion

This study identified that a decreased PLR, which was accompanied by a lower level of platelets but a higher level of lymphocytes, was associated with higher odds of ICU trauma patient mortality outcome. This marker was explored in addition to the conventional risk factors for mortality, such as male gender, old age, pre-existing HTN, CAD and ESRD, lower GCS, higher ISS, lower RBC level, and elevated aPTT. A study of the early WBC mobilization response during blood loss revealed that a relative leucocytosis was elicited by the central hypovolemia [24]. Therefore, a higher level of lymphocytes in non-survivors may also be recognized as an indicator of hypovolemia, implying the early and intensive fluid supplementation at the accident place is essential for the survival of severely injured patients. In addition, our interpretation that elevated lymphocyte level indicates hypovolemia is supported by the observation that this effect was accompanied by a decrease in RBC levels. It should be noted that hypovolemia leads to a drop in arterial blood pressure. To limit this effect, the total peripheral resistance in organ circulation is increased. In the case of the heart and brain, local blood flow regulation is dominant, and therefore, disturbances in the blood flow through these organs are limited. However, there is a drastic decrease in blood flow through the kidneys and gastrointestinal tract. The digestive system and kidneys are especially sensitive to a reduction in organ blood flow and hypoxia. Previous experimental studies showed that exposure of gastric mucosa to potentially noxious factors resulted in limited damage, as long as adequate blood flow is maintained [25]. Damage to the gastric mucosa is associated with a decrease in blood flow through the gastric mucosa [26], whereas the protection and healing of gastric mucosa is associated with an increase in gastric blood flow [27,28,29]. A similar effect of blood flow organ integrity was observed in other organs of the digestive system such as the duodenum [30], colon [31], liver [31] and pancreas [32]. Disturbances to pancreatic blood flow and pancreatic ischemia play an essential role in the development of acute pancreatitis in different clinical settings [33], including hypovolemic shock [34]. In addition, small bowel hypoxia leads to gut barrier failure associated with bacterial translocation, systemic inflammation, and the development of multiple organ dysfunction syndrome [35]. In the kidney, trauma and hypovolemia lead to acute kidney injury (AKI) [36,37].The high incidence of AKI in trauma patients should lead to early identification of those at risk of AKI to establish a resuscitation strategy that aims at preventing AKI. Assessment of neutrophil gelatinase-associated lipocalin [38] and angiopoietin-2 [39,40] seems to be useful in early the detection of AKI in addition to the classic biochemical markers such as a decrease in diuresis and glomerular filtration rate and an increase in serum creatinine [41]. This study revealed that critically ill patients who did not survive had a significantly higher lymphocyte counts than the survivors. The lymphocyte level may increase in leukemia, lymphoma, and viral infections. Neither of these situations is involved in critically injuries, especially immediately following injuries. For this reason, the increase in lymphocyte counts in non-survivors probably is not a true but is instead a spurious increase due to a decrease in body fluid volume and plasma volume as an effect of blood loss resulting from trauma. Therefore, a higher level of lymphocytes in non-survivors should be recognized as an indicator of hypovolemia. This observation proves that early and intensive fluid supplementation at the accident location is essential for the survival of severely injured patients, and this management should be continued after the patients arrive at the hospital. Correct hypovolemia helps to maintain normal arterial blood pressure and organ blood flow, which prevents multi-organ injury and reduces the risk of death.

Furthermore, lymphocyte levels are related to multiple organ dysfunction syndromes in patients with traumatic injury [19,20]. Many studies have also demonstrated that severe injury results in the disruption of the coagulation system or coagulopathy in a significant number of patients [16,17,18,42]. The observed increase in PT, aPTT, and INR in dead patients should be recognized as a sign of consumptive coagulopathy. Moreover, severe trauma may also result in remarkable blood loss and may lead to platelet consumption [16,17,18]. Therefore, it was not surprising to find a higher level of lymphocytes, a lower level of platelets, and a deduced lower PLR were associated with a higher risk of death in trauma patients with critical illness in this study. In the trauma patients admitted to the ICU, it has been well-recognized that the presence of acute kidney injury was associated with the increased mortality of the patients [43]. Therefore, in this study it is not surprising to find a significant increase in the serum level of BUN and Cr, the markers of kidney injury, in the dead patients than in the survivors. Notably, this study also revealed that, among the independent variables associated with risk for mortality, ESRD was associated with the highest odds of mortality risk.

However, some studies have reported different findings in trauma patients. In this study, no association was found between NLR and mortality outcome, while Wang et al. reported that the NLR is a useful biomarker for predicting isolated tibial plateau fracture severity in young and middle-aged adults [44]. Furthermore, NLR was also an independent predictor of mortality in ICU patients after major cardiac and vascular cancer surgical procedures [45,46]. It was reported that NLR is a very good independent predictor of lethal outcome in critically ill patients in the ICU, while the nature of the bacteremia influenced MLR and PLR [47]. We believe that the difference between this study’s results and the other studies was attributed to the time blood drawing for further analysis and the study patient population. In this study, the analyzed blood was drawn at the time of arrival at the emergency room but not during hospitalization or after a major operation; therefore, the time to incur a change in the neutrophils or monocytes in the circulation may not be sufficient. In addition, the study population included trauma patients with critical illness admitted to the ICU, but not patients with trauma injury to a specific body region; therefore, the platelet consumption following blood loss may be more dominant in the patients in this study. Since the lower levels of platelets and PLR were associated with the higher mortality of the patients with major trauma, the rapid attainment of high platelets/packed RBC ratio after the injury may be associated with significantly lower mortality for these trauma patients [48,49], albeit the optimal ratio of platelets to packed RBC in trauma patients requiring massive transfusion is still controversial.

Among various prediction models for the mortality outcomes of trauma patients, the trauma and injury severity score (TRISS) remains the most commonly used algorithm [50,51,52,53,54]. The TRISS calculator determines the probability of survival with age as a confounding variable, ISS (an anatomical variable), Revised Trauma Score (RTS, a physiological variable, a weighted summation of the coded variable values of the patient’s initial GCS score, systolic blood pressure (SBP), and respiratory rate (RR) [8]), and the use of different coefficients for blunt and penetrating injuries [55]. In this study, the inclusion of PLR in the algorithm for predicting mortality found 0.89 AUC with the accuracy, sensitivity, and specificity of 83.3%, 79.7%, and 83.8%, respectively. This predictive estimate is compatible with the TRISS calculated for trauma patients in the ICU (AUC = 0.88) [54], but less than that calculated for trauma patients (AUC = 0.93) [53]. Notably, the TRISS was originally developed for trauma patients but not for trauma patients in the ICU. The inclusion of PLR in the algorithm for mortality prediction in the study presents a predictive performance similar to that of the TRISS and including physiological change in the predictive algorithm. 

This study had some limitations. First, selection bias may exist in the retrospective design of the study, especially after excluding incomplete data. Second, the data of patients who died upon arrival to our ER were not recorded, and only in-hospital mortality, but not long-term mortality, was evaluated. Third, the platelets and WBC subtypes may fluctuate dynamically during the treatment course and may interfere with resuscitation procedures. Although blood was drawn upon patient arrival to the emergency room in this study, blood transfusion was less likely to occur. However, the fluid challenge did not adhere to the specific protocol, and the amount of infused fluid was unknown, which may have led to a bias in the outcome measurement. Further, the final cause of mortality was not registered in the trauma data, thus precluding further analysis. Finally, the population in this study was limited to a single urban trauma center, and the results may not be generalizable to other regions.

## 5. Conclusions

This study revealed that a higher level of lymphocytes, a lower level of platelets, and a lower PLR were associated with higher risk of death in trauma patients in the ICU.

## Figures and Tables

**Table 1:** General characteristic and injury severity of the dead and surviving patients.

Variables	Death *n* = 330		Survival *n* = 2524		OR (95% CI)		*p*
Sex							0.031
Male, *n* (%)	229	(69.4)	1599	(63.4)	1.31	(1.02–1.68)	
Female, *n* (%)	101	(30.6)	925	(36.6)	0.76	(0.60–0.98)	
Age (years)	63.3	±18.4	56.6	±19.2	—		<0.001
Comorbidities							
CVA, *n* (%)	20	(6.1)	134	(5.3)	1.15	(0.71–1.87)	0.570
HTN, *n* (%)	147	(44.5)	873	(34.6)	1.52	(1.21–1.92)	<0.001
CAD, *n* (%)	44	(13.3)	166	(6.6)	2.19	(1.53–3.11)	<0.001
CHF, *n* (%)	3	(0.9)	13	(0.5)	1.77	(0.50–6.25)	0.367
DM, *n* (%)	72	(21.8)	479	(19.0)	1.19	(0.90–1.58)	0.219
ESRD, *n* (%)	32	(9.7)	55	(2.2)	4.82	(3.07–7.58)	<0.001
GCS, median (IQR)	5	(3–12)	15	(11–15)	—		<0.001
3–8, *n* (%)	222	(67.3)	468	(18.5)	9.03	(7.03–11.61)	<0.001
9–12, *n* (%)	26	(7.9)	319	(12.6)	0.59	(0.39–0.90)	0.013
13–15, *n* (%)	82	(24.8)	1737	(68.8)	0.15	(0.12–0.20)	<0.001
ISS, median (IQR)	25	(25–29)	17	(16–24)	—		<0.001
1–15, *n* (%)	22	(6.7)	509	(20.2)	0.28	(0.18–0.44)	<0.001
16–24, *n* (%)	58	(17.6)	1426	(56.5)	0.16	(0.12–0.22)	<0.001
≥25, n (%)	250	(75.8)	589	(23.3)	10.27	(7.85–13.42)	<0.001
RBCs (millions/μL)	3.9	±0.9	4.3	±0.8			<0.001
Monocytes (count/μL)	631	±450	626	±415	—		0.834
Neutrophils (count/μL)	9725	±5550	10,198	±53,932	—		0.136
Platelets (count/μL)	175,842	±61,713	206,890	±69,006	—		<0.001
Lymphocytes (count/μL)	2458	±1940	1971	±1453	—		<0.001
MLR	0.4	±0.5	0.5	±0.5	—		0.605
NLR	7.6	±8.5	8.2	±7.8	—		0.211
PLR	124.3	±110.3	150.6	±106.5	—		<0.001
PT (s)	13.3	±6.3	11.2	±3.1	—		<0.001
aPTT (s)	33.0	±13.5	26.9	±4.8	—		<0.001
INR	1.3	±0.6	1.1	±0.2	—		<0.001
AST (U/L)	87.8	±152.2	79.8	±170.6	—		0.418
ALT (U/L)	50.3	±91.9	53.0	±93.8	—		0.626
BUN (mg/dL)	22.0	±18.0	16.4	±11.2	—		<0.001
Cr (mg/dL)	1.9	±2.3	1.1	±1.5	—		<0.001
Hospital stay (days)	11.1	±15.2	7.7	±9.8			<0.001

aPTT, activated partial thromboplastin time; ALT, alanine aminotransferase; AST, aspartate aminotransferase; BUN, blood urea nitrogen; CAD, coronary artery disease; CHF, congestive heart failure; CI, confidence interval; Cr, creatinine; CVA, cerebral vascular accident; DM, diabetes mellitus; ESRD, end-stage renal disease; GCS, Glasgow Coma Scale; HTN, hypertension; IQR, interquartile range; INR, international normalized ratio; ISS, injury severity score; MLR, monocyte to lymphocyte ratio; NLR, neutrophil-to-lymphocyte ratio; OR, odds ratio; PLR, platelet-to-lymphocyte ratio; PT, prothrombin time.

**Table 2:** Univariate and multivariate logistic regression analysis of the independent risk factors for mortality outcome.

Variables	Univariate Analysis			Multivariate Analysis		*p*
	OR (95%CI)		*p*	OR (95% CI)		
Male	1.31	(1.02–1.68)	0.032	1.45	(1.05–2.01)	0.024
Age	1.02	(1.01–1.03)	<0.001	1.03	(1.02–1.04)	<0.001
HTN	1.52	(1.21–1.92)	<0.001	1.11	(0.80–1.54)	0.538
CAD	2.19	(1.53–3.11)	<0.001	1.73	(1.09–2.75)	0.020
ESRD	4.82	(3.07–7.58)	<0.001	3.12	(1.32–7.37)	0.010
GCS	0.77	(0.75–0.79)	<0.001	0.79	(0.76–0.82)	<0.001
ISS	1.11	(1.09–1.13)	<0.001	1.06	(1.04–1.08)	<0.001
RBC	0.53	(0.46–0.61)	<0.001	0.80	(0.66–0.98)	0.032
Lymphocytes	1.19	(1.12–1.27)	<0.001	1.31	(1.16–1.48)	<0.001
Platelets	0.48	(0.40–0.58)	<0.001	0.54	(0.41–0.67)	<0.001
PLR	0.97	(0.96–0.98)	<0.001	0.98	(0.98–0.99)	0.024
PT	1.11	(1.08–1.15)	<0.001	1.03	(0.98–1.07)	0.235
aPTT	1.11	(1.08–1.13)	<0.001	1.06	(1.03–1.09)	<0.001
INR	3.92	(2.64–5.81)	<0.001	1.09	(0.61–1.95)	0.779
BUN	1.03	(1.02–1.03)	<0.001	1.02	(1.00–1.03)	0.054
Cr	1.20	(1.13–1.27)	<0.001	1.02	(0.91–1.14)	0.705

aPTT, activated partial thromboplastin time; ALT, alanine aminotransferase; AST, aspartate aminotransferase; BUN, blood urea nitrogen; CAD, coronary artery disease; CI, confidence interval; Cr, creatinine; ESRD, end-stage renal disease; GCS, Glasgow Coma Scale; HTN, hypertension; INR, international normalized ratio; ISS, injury severity score; OR, odds ratio; PLR, platelet-to-lymphocyte ratio; PT, prothrombin time.

## Abbreviations

ALT	alanine aminotransferase
Aptt	activated partial thromboplastin time
AST	aspartate aminotransferase
BUN	blood urea nitrogen
CAD	coronary artery disease
CBC	complete blood count
CHF	congestive heart failure
CI	confidence interval
Cr	creatinine
CVA	cerebrovascular accident
DM	diabetes mellitus
ESRD	end-stage renal disease
GCS	Glasgow Coma Scale
HTN	hypertension
ICU	intensive care unit
INR	international normalized ratio
IQR	interquartile range
ISS	Injury Severity Score
MLR	monocyte-to-lymphocyte ratio
NLR	neutrophil-to-lymphocyte ratio
PLR	platelet-to-lymphocyte ratio
PT	prothrombin time
RBCs	red blood cells
WBCs	white blood cells
